# Neutrophil-to-lymphocyte ratio predicts parenchymal hematoma after mechanical thrombectomy in basilar artery occlusion

**DOI:** 10.3389/fneur.2022.920349

**Published:** 2022-10-06

**Authors:** Yonggang Hao, Zhizhou Hu, Xiurong Zhu, Zhao Chen, He Jiang, Yang Lei, Jiasheng Liao, Kefeng Lv, Kuiyun Wang, Hongjun Wang, Jiachuan Liao, Wenjie Zi, Shunfu Jiang, Chong Zheng

**Affiliations:** ^1^Department of Neurology, Dushu Lake Hospital Affiliated to Soochow University, Suzhou, China; ^2^Department of Neurology, Longyan First Affiliated Hospital of Fujian Medical University, Longyan, China; ^3^Department of Neurology, Chongzhou People's Hospital, Chongzhou, China; ^4^Department of Neurology, Yaan People's Hospital, Yaan, China; ^5^Department of Neurology, The First People's Hospital of Neijiang, Neijiang, China; ^6^Department of Neurology, Wulong District People's Hospital, Chongqing, China; ^7^Department of Neurology, Suining No.1 People's Hospital, Suining, China; ^8^Department of Neurology, Dongguan People's Hospital, Dongguan, China; ^9^Department of Neurology, The Jintang First People's Hospital, Jintang, China; ^10^Department of Neurology, Fengdu People's Hospital, Fengdu, China; ^11^Department of Neurology, Santai County People's Hospital of North Sichuan Medical College, Santai, China; ^12^Department of Neurology, Xinqiao Hospital and the Second Affiliated Hospital, Army Medical University (Third Military Medical University), Chongqing, China; ^13^Department of Neurology, Jingdezhen First People's Hospital, Jingdezhen, China

**Keywords:** intracranial hemorrhage, endovascular treatment, thrombectomy, basilar artery occlusion, ischemic stroke

## Abstract

**Background:**

parenchymal hematoma (PH) is a severe complication of endovascular treatment (EVT) for acute basilar artery occlusion (ABAO). This study aimed to evaluate the incidence and predictors of PH after EVT for ABAO.

**Methods:**

Using data from the Endovascular Treatment for Acute Basilar Artery Occlusion Study, we enrolled patients treated with mechanical thrombectomy from the BASILAR registry. PH was assessed in accordance with the Heidelberg Bleeding Classification. Logistic regression was used to identify predictors of PH.

**Results:**

A total of 639 patients were included. Forty-eight patients (7.5%) were diagnosed with PH within 48 h of EVT. Ninety-day mortality was higher in patients with PH compared with those without (81.3 vs. 42.8%, *P* < 0.001). Favorable neurological outcomes (modified Rankin scale score, 0–3) rates was lower in patients with PH compared with those without (6.3 vs. 34.5%, *P* < 0.001). With a multivariate analysis, hypertension [odds ratio (OR) = 2.30, 95% confidence interval (CI) 1.04–5.08], pre-treatment National Institutes of Health Stroke Score (NIHSS, >25; OR = 3.04, 95% CI 1.43–6.45), and Neutrophil-to-lymphocyte ratio (NLR, >10; OR = 1.88, 95% CI 1.02–3.48) were associated with PH after EVT.

**Conclusions:**

PH occurred at a rate of 7.5% after EVT in patients with ABAO. Hypertension, higher baseline NIHSS, and higher NLR value increase the risk of PH after EVT for ABAO.

## Introduction

Acute basilar artery occlusion (ABAO) is associated with high morbidity and mortality rates (1, 2). Given the benefits of endovascular treatment (EVT) in patients with acute ischemic stroke caused by large artery occlusion in the anterior circulation (3), most patients with ABAO undergo EVT. Despite lack of data from randomized clinical trials, several large-sample retrospective and prospective studies have indicated that EVT may be reasonable for carefully selected patients with acute ischemic stroke due to ABAO (4, 5). Intracranial hemorrhage (ICH) is a common complication of EVT. In particularly, parenchymal hematoma (PH) is associated with high morbidity and mortality. Predictors of PH after large artery occlusion in the anterior circulation in patients treated with EVT have been extensively studied (6, 7). Conversely, research to identify predictors of PH after ischemic stroke in the posterior circulation is scarce. Therefore, identifying predictors of PH after EVT for patients with acute ischemic stroke duo to ABAO in real-world practice is important to continuously improve the efficacy of EVT. Using data from the Endovascular Treatment for Acute Basilar Artery Occlusion Study (BASILAR) (5), a multicenter registry program in China, we analyzed potential predictors of PH after EVT in patients with acute ischemic stroke caused by ABAO.

## Methods and materials

### Patients

Patients were enrolled from BASILAR, which is a nationwide prospective registry of consecutive patients who presented with an acute, symptomatic, radiologically confirmed BAO in 47 comprehensive stroke centers across 15 provinces in China. Details of the BASILAR registry can be found elsewhere (5). Briefly, the BASILAR registry included patients with acute ischemic stroke due to ABAO who underwent EVT from January 2014 to March 2019.

Endovascular treatment was proposed if patients (1) were aged ≥18 years; (2) were diagnosed with acute ischemic stroke due to ABAO or distal intracranial vertebral artery (V4 segment) occlusion resulting in occluded flow to the basilar artery; (3) underwent standard medical treatment plus EVT within 24 h of stroke onset; and (4) had a pre-stroke modified Rankin scale (mRS) score of <2. Patients were excluded if they (1) had evidence of concomitant ICH or severe gastrointestinal bleeding; (2) had a serious, advanced, or terminal illness; and (3) were pregnant or lactating.

Datails of the pre-procedure imaging evaluation, endovascular procedures and post-procedure evaluation have been reported previously (5). Successful recanalization was defined as a modified Treatment in Cerebral Infarction (mTICI) score of 2b or 3. Rescue therapies, including balloon angioplasty, stent implantation, intra-arterial thrombolysis, or intra-catheter tirofiban administration (8), were performed if recanalization of the target artery failed. Intracranial hemorrhage was diagnosed and classified according to Heidelberg Bleeding Classification (9).

### Post-procedure evaluation

CT was usually performed 24 h after the procedure, or whenever ICH was indicated by clinical symptoms. All CT, MR angiography, and digital subtraction angiography images were sent to the core lab at Xinqiao Hospital. Two neuroradiologists (W. Liu and W. Huang) who were blinded to clinical outcomes evaluated the results independently for imaging determinations. In case of disagreement, a third experienced neuroradiologist (Z. Shi) was invited to make the final decision. ICH was classified as one of the following subtype: Hemorrhagic infarction (HI) 1: scattered small petechiae, no mass effect; HI2: confluent petechiae, no mass effect; Parenchymal hematoma (PH) 1: hematoma within infarct tissue and occupied less than 30% of the infarct volume, no substantive mass effect; PH2: hematoma occupied 30% or more of the infarct volume, with obvious mass effect; Remote parenchymal hematoma (rPH): parenchymal hematoma remote from the infarct tissue; Intraventricular hemorrhage (IVH); Subarachnoid hemorrhage (SAH); Subdural hemorrhage (SDH) in accordance with the Heidelberg Bleeding Classification (9). PH1, PH2 and rPH were categorized as PH in present study. Ninety days after EVT, the mRS was used to assess patients during a clinic visit. A favorable neurological outcome was defined as an mRS score of 0–3.

### Statistical analysis

Continuous variables were analyzed with the Student's *t*-test or the Mann–Whitney *U*-test depending on the normality of distribution. Categorical variables were analyzed with the Chi-squared test or Fisher's exact test. A multivariate logistic regression analysis was applied to estimate independent predictors of PH after EVT in patients with acute ischemic stroke caused by ABAO. Entered factors were those with at least marginal significance (*P* < 0.1) with a univariate analysis. The statistical analysis was performed using SPSS 23.0 (IBM, Armonk, NY).

## Results

**Figure 1:**
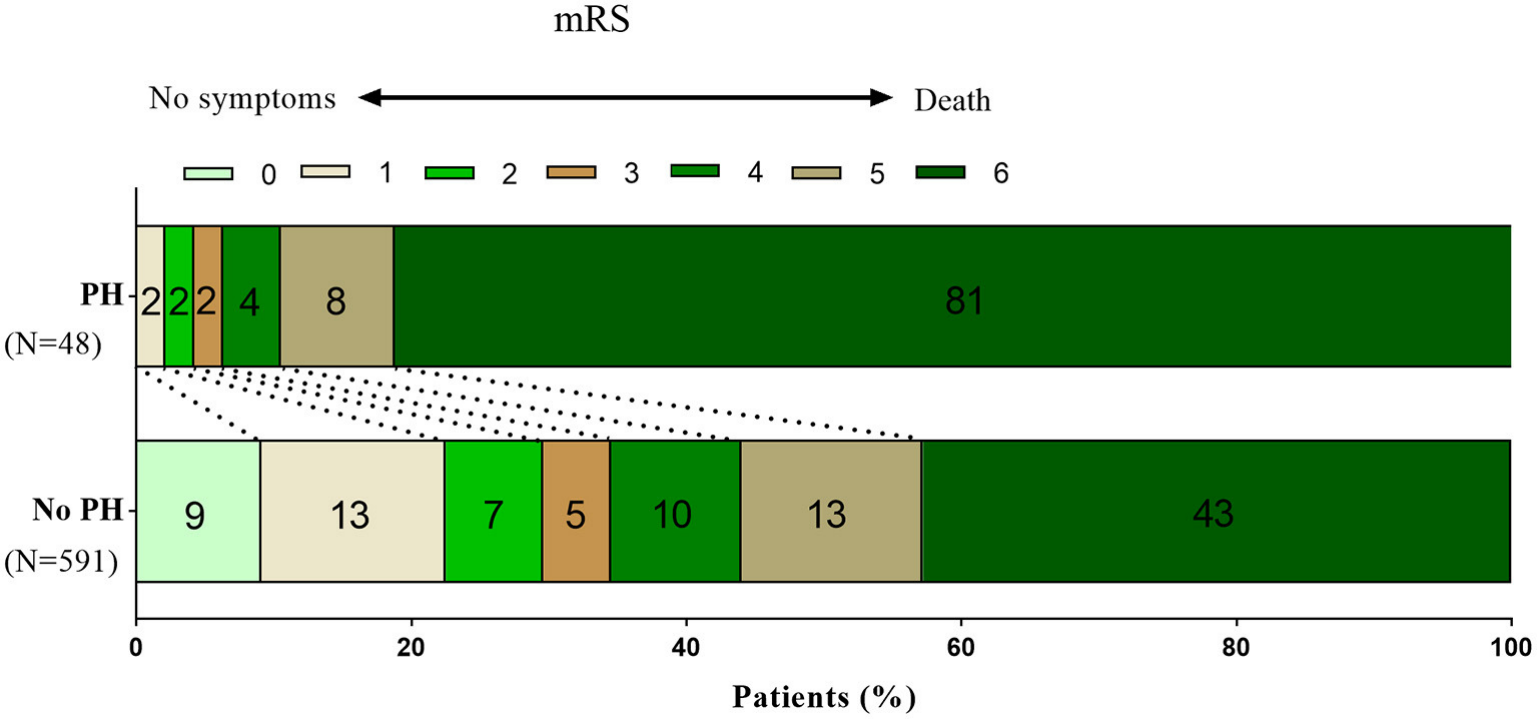
Distribution of mRS scores at 90 days in patients with and without PH. Fewer patients with PH reached a favorable outcome (mRS0–3) at 90 days after the index stroke than patients without PH (6.3 vs. 34.5%, *P* < 0.001). Ninety-day mortality was higher in patients with PH than in patients without PH (81.3 vs. 42.8%, *P* < 0.001). mRS, modified Rankin scale; PH, parenchymal hematoma.

**Figure 2:**
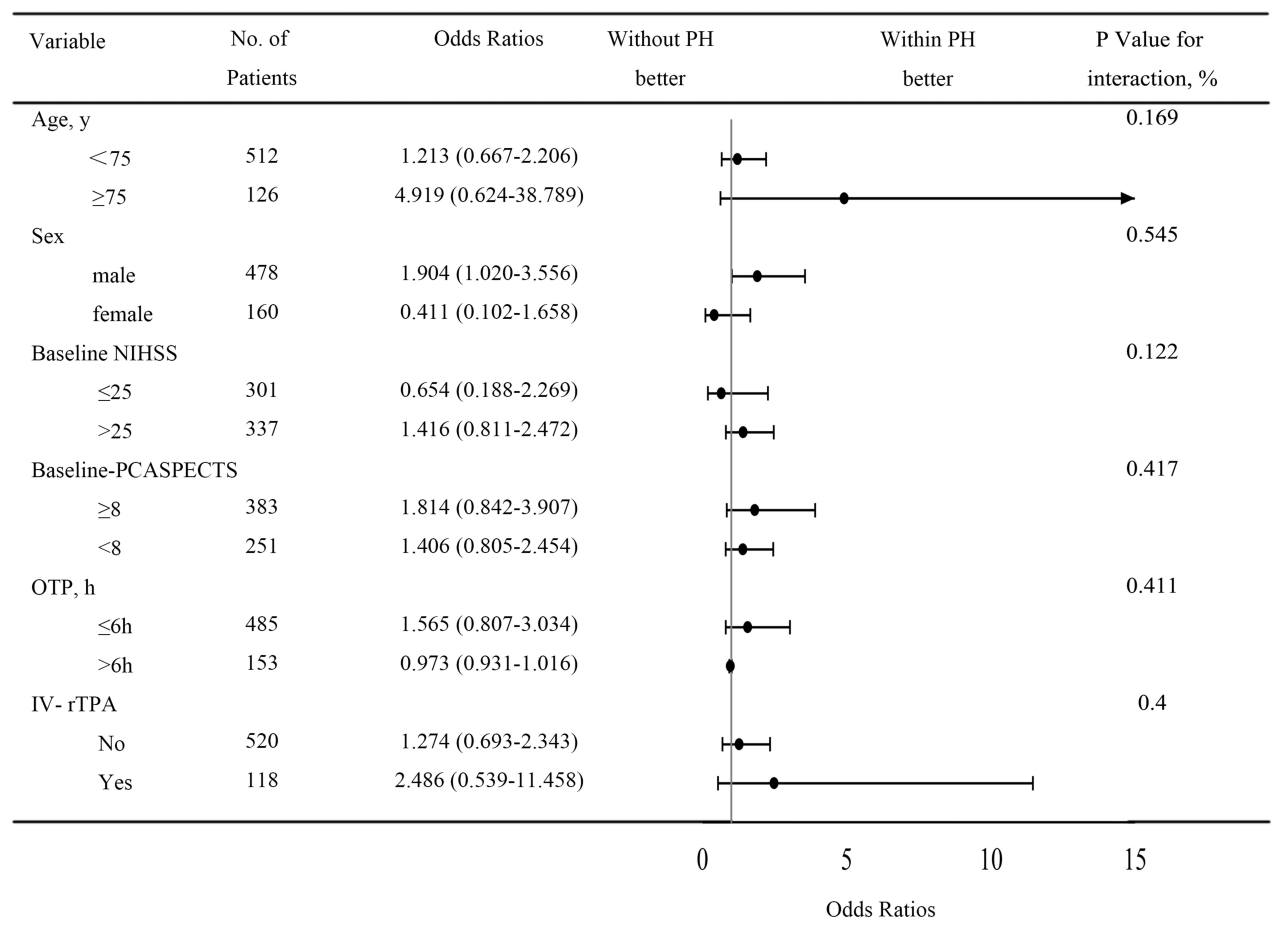
Subgroup analysis of PH after intra-arterial tirofiban treatment. Intra-arterial tirofiban administration did not increase the risks of PH. CI, confidence interval; OR, relative risk; NIHSS, National Institute of Health Stroke Scale; PCASPECTS, Posterior circulation Alberta Stroke Program Early Computed Tomography Score; OTP, symptoms onset to puncture time; IV-rTPA, intravenous recombinant tissue plasminogen activator.

A total of 654 patients were treated with MT. Eight patients were excluded from the study due to missing follow-up information on outcomes at 90 days, seven patients were excluded because of arterial perforation occurred during the procedure, and 639 patients were included in the final analysis. Successful recanalization (mTICI score, 2b/3) was achieved in 520 of 639 patients (81.4%), and 207 patients (32.4%) achieved a favorable functional outcome (mRS score, 0–3). PH was observed in 48 patients (7.5%) within 48 h after EVT. Ninety-day mortality was higher in patients with PH compared with patients without PH (81.3 vs. 42.8%, respectively; *P* < 0.001; Figure 1). With a 90-day mRS score of 0–3, a favorable neurological outcome was proportionally less likely in patients with PH compared with those without PH (6.3 vs. 34.5%, respectively; *P* < 0.001; Figure 1). The baseline characteristics of patients according to PH are presented in Table 1. With a univariate analysis, the prevalence of hypertension in patients with PH was higher than that in patients without (83.3 vs. 68.9%, *P* = 0.034). Patients with PH had higher baseline National Institutes of Health Stroke Scale (NIHSS) scores (median, 31 vs. 26, respectively; *P* = 0.006) and higher neutrophil-to-lymphocyte ratio (NLR; median, 10.8 vs. 7.9, respectively; *P* = 0.040). Poorer collateral circulation (American Society of Interventional and Therapeutic Neuroradiology/ Society of Interventional Radiology score, <2) was more common in patients with PH than in patients without (82.2 vs. 58.2%, respectively; *P* < 0.001). The rate of successful recanalization (mTICI score, >2a) was 72.9% in patients with PH and 82.1% in patients without PH (*P* = 0.170). There was no significant difference in intra-arterial tirofiban administration between the group with PH and the group without PH (39.6 vs. 48.8%, respectively; *P* = 0.383). With a subgroup analysis, intra-arterial tirofiban administration did not increase the risk of PH (Figure 2).

**Table 1:** Baseline characteristics and outcomes of patients with and without PH.

**Characteristics**	**PH**		***P*-value**
	**With (*n* = 48)**	**Without (*n* = 591)**	
Age, median (IQR)	65 (58–72)	64 (56–73)	0.740
Male gender, *n* (%)	40 (83.3)	438 (74.1)	0.171
Hypertension, *n* (%)	40 (83.3)	407 (68.9)	0.034
Hyperlipidemia, *n* (%)	15 (31.3)	197 (33.3)	0.874
DM, *n* (%)	12 (25.0)	133 (22.5)	0.720
AF, *n* (%)	15 (31.3)	119 (20.1)	0.095
Previous cerebral infarction, *n* (%)	9 (18.8)	130 (22.0)	0.717
Previous TIA, *n* (%)	0 (0.0)	8 (1.4)	1.000
Anticogagulation	2 (4.3)	11 (1.9)	0.249
**Baseline measurements**			
SBP, mmHg, median (IQR)	157 (135–166)	150 (133–166)	0.384
PC-ASPECTS, median (IQR)	8 (7–9)	8 (7–9)	0.197
NIHSS score, median (IQR)	31 (27.0–34.0)	26 (16.0–33.0)	0.006
Serum glucose, mmol/L, median (IQR)	7.7 (6.2–9.9)	7.3 (6.1–9.6)	0.631
Baseline NLR, median (IQR)	10.8 (6.0–15.8)	7.9 (5.0–12.5)	0.040
**Stroke etiology**, ***n*** **(%)**			
LAA	29 (60.4)	384 (65.0)	0.725
CE	16 (33.3)	155 (26.2)	
SOE	1 (2.1)	17 (2.9)	
SUE	2 (4.2)	35 (5.9)	
**Occlusion site**, ***n*** **(%)**			
PCA	3 (6.3)	12 (2.0)	0.178
BA1	17 (35.4)	187 (31.6)	
BA2	9 (18.8)	184 (31.1)	
BA3	8 (16.7)	99 (16.8)	
V 4	11 (22.9)	109 (18.4)	
**Anesthesia**, ***n*** **(%)**			
GA	18 (38.3)	237 (40.6)	0.877
LA	29 (61.7)	347 (59.4)	
**Procedure process and results**			
ASITN/SIR, *n* (%)			
0~1	37 (82.2)	344 (58.2)	0.003
2	8 (17.8)	165 (27.9)	
3~4	0 (0.0)	82 (13.9)	
OTP, min, median (IQR)	368 (220.8–512.5)	320 (220.0–494.5)	0.410
OTR, min, median (IQR)	480 (362.3–680.5)	437 (324.5–620.8)	0.445
Passes of retriever >3, *n* (%)	3 (6.8)	29 (5.1)	0.866
Intravenous thrombolysis, *n* (%)	10 (20.8)	128 (21.7)	1.000
Intra-arterial thrombolysis, *n* (%)	10 (20.9)	69 (11.7)	0.108
Intra-arterial tirofiban, *n* (%)	19 (39.6)	288 (48.8)	0.383
mTICI, 2b or 3, *n* (%)	35 (72.9)	485 (82.1)	0.170
90d mRS ≤3, *n* (%)	3 (6.3)	204 (34.5)	< 0.001
90d Mortality, *n* (%)	39 (81.3)	253 (42.8)	< 0.001

PH, parenchymal hematoma; IQR, interquartile range; DM, diabetic mellitus; AF, atrial fibrillation; TIA, transient ischemic attack; SBP, systolic blood pressure; PC-ASPECTS, Posterior circulation Alberta Stroke ProgramEarly Computed Tomography Score; NIHSS, National Institute of Health Stroke Scale; NLR, Neutrophil-to-lymphocyte ratio; LAA, large-artery atherosclerosis; CE, Cardioembolism; SOE, Stroke of other determined cause; SUE, Stroke of undetermined cause; PCA, posterior cerebral artery; BA, basilar artery; V4, vertebral artery V4 segment; GA, general anesthesia; LA, lacal anesthesia; ASITN/SIR, American Society of Interventional and Therapeutic Neuroradiology/Society of Interventional Radiology; OTP, symptoms onset to puncture time; OTR, symptoms onset to recanalization time; mTICI, modified Thrombolysis in Cerebral Infarction; mRS, modified Rankin scale.

With a multivariate logistic analysis, hypertension [odds ratio (OR) = 2.30, 95% confidence interval (CI) 1.04–5.08], baseline NIHSS score >25 (OR = 3.04, 95% CI 1.43–6.45), and baseline NLR > 10 (OR = 1.88, 95% CI 1.02–3.48) were associated with PH after EVT (Table 2).

**Table 2:** Multivariate analysis of predictors of PH after endovascular treatment.

**Risk factors**	**Increment/categories**	**OR (95% CI)**	***P-*value**
Hypertension	vs. no	2.30 (1.04–5.08)	0.039
AF	vs. no	1.58 (0.81–3.06)	0.178
Baseline NIHSS score >25	vs. ≤25	3.04 (1.43–6.45)	0.004
NLR >10	vs. ≤10	1.88 (1.02–3.48)	0.043
ASITN/SIR 0–1	vs. ≥2	0.67 (0.32–1.39)	0.284
mTICI 0–2a	vs. ≥2b	0.68 (0.34–1.35)	0.269

PH, parenchymal hematoma; OR, odds ratio; CI, interval confidence; AF, atrial fibrillation; NIHSS, National Institute of Health Stroke Scale; NLR, neutrophil-to-lymphocyte ratio; ASITN/SIR, American Society of Interventional and Therapeutic Neuroradiology/Society of Interventional Radiology; mTICI, modified Thrombolysis in Cerebral Infarction.

## Discussion

In this multi-center registry study, which may reflect real-world practices, we observed an PH incidence of 7.5%, which is higher than observations in randomized controlled trials that reported in the Basilar Artery Occlusion Endovascular Intervention vs. Standard Medical Treatment (BEST; 3.0%) (10). PH was associated with less favorable neurological outcomes and an increased risk of mortality at 90 days. These observations are consistent with anterior circulation large artery occlusion (7). A history of hypertension, a higher baseline NIHSS score and baseline NLR >10 were independent predictors of PH after EVT in patients with ABAO.

The incidence of PH in our research was lower than the recent publications with a frequency of 16.8% (11), but higher compared with that reported in other Asian studies on ABAO (4.2%) (4). These differences might result from the heterogeneous treatment experiences of nationwide centers. The rate of PH in the present study was lower compared with randomized clinical trial (12) and other registry study (7) that have investigated anterior circulation large artery occlusion. The vertebral basilar system has a relatively high proportion of white matter, and ICH after ischemic stroke tends to occur in areas of gray matter where capillary density is greater, such as the deep gray nuclei and the cerebral cortex (13). Abnormal blood–brain barrier (BBB) permeability resulting from ischemic capillary endothelial dysfunction may lead to ICH (14).

In this study, we showed that admission NLR >10 is associated with PH after EVT in patients with ABAO. Results of others studies in patients treated with EVT (15, 16) suggested that elevated inflammatory markers, including NLR, might predict a poor outcome. However, the underlying mechanisms by which neutrophils contribute to poor outcome remain uncertain. BBB disruption occurs early after stroke and facilitates the infiltration of peripheral inflammatory cells into the infarct tissue. By increasing local inflammation, this process may potentiate further endothelial damage leading to additional brain injury from hemorrhagic transformation. Other investigators have suggested that a higher rate of hemorrhagic transformation, which is a sign of endothelial disruption, is associated with brain leukocyte infiltration (17). Moreover, neutrophile granulocytes have been shown as an important source of MMP-9 that can cause early disruption of the BBB in ischemic stroke (18). BBB disruption has been suggested as an underlying mechanism for intracranial hemorrhage after ischemic stroke (19).

Previous literature has depicted age, a history of hypertension, stroke severity, a poor collateral circulation, delayed recanalization treatment, and multiple passes with thrombectomy devices as predictors of PH (7, 20), which is partly similar to present results. Safety of tirofiban in patients with acute ischemic stroke have been investigated in various studies. In our study, intra-arterial tirofiban administration as a rescue therapy was not associated with PH. Neuberger et al. concluded that intravenous bolus and maintenance for 48-h administration of tirofiban during the course of MT for ABAO predicts any ICH and SICH occurrence (11). Our finding is consistent with other clinical trial that intra-arterial tirofiban administration was not associated with increased risk of ICH in patients with ischemic stroke due to large artery occlusion in anterior cerebral circulation (8).

Finally, another parameter frequently used to assess safety in EVT studies is the occurrence of symptomatic Intracranial hemorrhage (SICH), defined by an increase of the NIHSS compared with baseline. We chose to report the rate and predictors of PH rather than SICH for several reasons. The NIHSS has a good correlation with the middle cerebral artery territory size infarct but underestimates clinical severity in posterior circulation stroke (21). In other words, the NIHSS may be limited in its ability to measure the clinical deterioration of patients with posterior circulation cerebral infarction. For example, patients with a severe stroke and a high baseline NIHSS (>20–25) can rarely further increase their NIHSS score.

Several limitations of the present study should be emphasized when interpreting the results. As the study adopted a prospective observational design, we did not assess other potentially relevant variables, such as peri-procedural blood pressure variations, heparin use during MT, and anti-platelet regimes, which may influence the risk of ICH.

## Conclusions

In conclusion, our study shows that the incidence of PH after EVT in patients with ABAO is similar to that of patients with anterior circulation large vessel occlusion in real-world practice. A history of hypertension, a higher baseline NIHSS score and baseline NLR >10 may increase the risk of PH after EVT in patients with ABAO.

## Data availability statement

The raw data supporting the conclusions of this article will be made available by the authors, without undue reservation.

## Ethics statement

The studies involving human participants were reviewed and approved by the Ethics Committee of the Xinqiao Hospital (Second Affiliated Hospital), Army Medical University Board (201308701). The patients/participants provided their written informed consent to participate in this study.

## Author contributions

YH, XZ, and ZH interpreted the data and drafted the manuscript. CZ and SJ contributed to the conception and design of the study. ZC, HJ, and YL did the statistical analyses. JSL, KL, and KW performed acquisition, and analysis or interpretation of data. HW and JCL provided technical or material support and made critical revision of the manuscript. All authors contributed to the article and approved the submitted version.

## Funding

This work was supported by Army Medical University Clinical Medical Research Talent Training Program (No. 2019XLC2008, 2019XLC3016, 2018XLC2013, and 2018XLC3039), National Science Fund for Distinguished Young Scholars (No. 81525008), Chongqing Major Disease Prevention and Control Technology Research Project (No. 2019ZX001), Major Clinical Innovation Technology Project of the Second Affiliated Hospital of the Army Military Medical University (No. 2018JSLC0017), and Zhejiang Provincial Natural Science Found (No. LY19H090016).

## Conflict of interest

The authors declare that the research was conducted in the absence of any commercial or financial relationships that could be construed as a potential conflict of interest.

## Publisher's note

All claims expressed in this article are solely those of the authors and do not necessarily represent those of their affiliated organizations, or those of the publisher, the editors and the reviewers. Any product that may be evaluated in this article, or claim that may be made by its manufacturer, is not guaranteed or endorsed by the publisher.
